# Comprehensive Gene Expression Profiling Analysis of Adipose Tissue in Male Individuals from Fat- and Thin-Tailed Sheep Breeds

**DOI:** 10.3390/ani13223475

**Published:** 2023-11-10

**Authors:** Sana Farhadi, Karim Hasanpur, Jalil Shodja Ghias, Valiollah Palangi, Aristide Maggiolino, Vincenzo Landi

**Affiliations:** 1 Department of Animal Science, Faculty of Agriculture, University of Tabriz, Tabriz 51666-16471, Iran; farhadiso16@tabrizu.ac.ir (S.F.); shodja@tabrizu.ac.ir (J.S.G.); 2 Department of Animal Science, Faculty of Agriculture, Ege University, 35100 Izmir, Türkiye; valiollah.palangi@ege.edu.tr; 3 Department of Veterinary Medicine, University of Bari Aldo Moro, 70010 Valenzano, Italy; vincenzo.landi@uniba.it

**Keywords:** fat deposition, fat-tailed sheep, machine learning, RNA-seq

## Abstract

**Simple Summary:**

For this paper, we investigated the differences in adipose tissue deposition between sheep breeds with fat and thin tails, relying on advanced techniques like meta-analyses and machine learning to analyze gene expression data. Our findings revealed key genes associated with fat metabolism, shedding light on the genetic factors influencing tail fat in sheep. Notably, three specific genes (*POSTN*, *K35*, and *SETD4*) were identified as significant biosignatures related to fat deposition. This innovative approach (combining data analysis and machine learning) enhances our understanding of how to optimize fat deposition in sheep breeds, which holds potential for more efficient animal breeding strategies and carcass fat reduction.

**Abstract:**

It has been shown that tail fat content varies significantly among sheep breeds and plays a significant role in meat quality. Recently, significant efforts have been made to understand the physiological, biochemical, and genomic regulation of fat deposition in sheep tails in order to unravel the mechanisms underlying energy storage and adipose tissue lipid metabolism. RNA-seq has enabled us to provide a high-resolution snapshot of differential gene expression between fat- and thin-tailed sheep breeds. Therefore, three RNA-seq datasets were meta-analyzed for the current work to elucidate the transcriptome profile differences between them. Specifically, we identified hub genes, performed gene ontology (GO) analysis, carried out enrichment analyses of the Kyoto Encyclopedia of Genes and Genomes (KEGG) pathways, and validated hub genes using machine learning algorithms. This approach revealed a total of 136 meta-genes, 39 of which were not significant in any of the individual studies, indicating the higher statistical power of the meta-analysis. Furthermore, the results derived from the use of machine learning revealed *POSTN*, *K35*, *SETD4*, *USP29*, *ANKRD37*, *RTN2*, *PRG4*, and *LRRC4C* as substantial genes that were assigned a higher weight (0.7) than other meta-genes. Among the decision tree models, the Random Forest ones surpassed the others in adipose tissue predictive power fat deposition in fat- and thin-tailed breeds (accuracy > 0.85%). In this regard, combining meta-analyses and machine learning approaches allowed for the identification of three important genes (*POSTN*, *K35*, *SETD4*) related to lipid metabolism, and our findings could help animal breeding strategies optimize fat-tailed breeds’ tail sizes.

## 1. Introduction

Sheep are the leading meat and wool producers [1,2], with 20–25% of their world population being fat-tailed [3,4]. These sheep were first recorded on an Uruk III stone vessel about 5000 years ago [5]. These breeds are used in the different lamb production systems that are currently adopted around the world, reflecting different breeders’ economic conditions, consumers’ preferences, resources, and production aims. However, traditionally, sheep breeding is chiefly based on dairy breeds for both milk and meat production [2], with lamb being considered a high-quality product and even a delicacy in many countries [6].

In several breeds, artificial and natural selection have indirectly led to the development of adaptation to varying environmental conditions in different geographic regions. Within this spectrum, fat-tailed sheep are a noteworthy category of the world sheep population [7]. These sheep are primarily found in the Middle East, North and East Africa, and Central Asia. As highlighted by Xu et al. [8], fat tails serve as vital energy reserves that are crucial for survival in the wake of challenging conditions like droughts and food scarcity. This notion was further affirmed by Mwacharo et al. [9], who underscored that fat-tailed sheep predominate in the deserts and highlands of northern Africa, as well as in the semi-arid and arid regions of eastern and southern Africa.

The level of lipid storage in the carcass influences meat quality [10,11]. Moreover, fat affects many physical and chemical properties (e.g., color, water holding capacity) that are fundamental in the purchasing decision process [12,13,14,15]. Also, considering the increase in human living standards, people prefer tasty and healthy meat. Hence, increasing attention has been paid to provide leaner meat and to produce meat with intermuscular fat characterized by a lower saturation and higher unsaturation of fatty acids [16]. Adipose tissue is an important storage location for excess energy [10], with tail and subcutaneous fat being domestic animals’ major fat storage sites [11]. The number of sheep breeds that have evolved worldwide is very high, with many being found specifically in northern Africa, the Middle East, Central Asia, and Western China [17]. It is assumed that the first home sheep were thin, but over time, due to the need to store energy for harsh environmental conditions, fat-tailed breeds gradually appeared [18]. However, in modern sheep industry systems, thin-tailed breeds are more desirable, while tail tissue has lost its importance in fat-tailed sheep. There are several logical reasons behind this trend: 1. in modern sheep breeding systems, there is no need for energy from tail tissue because intensive or semi-intensive feeding systems are preferred; 2. feed efficiency is decreased due to the higher energy requirement of fat anabolism as compared to the generation of protein or other molecules 3. today, the health of consumers is threatened by the consumption of high-fat foods; 4. a large tail can cause problems for mating and animal welfare. Therefore, raising thin-tailed sheep is cost-effective for both producers and consumers, and one of the sheep industry’s primary goals is to study lean meat. In this context, disentangling the molecular mechanism of fat accumulation is critical to reduce its content in the carcass, as the manipulation of fat deposition is crucial to produce lean meat.

To date, various genomic- [19,20,21,22,23,24,25] and transcriptomic-based studies [26,27,28,29,30] have aimed to pinpoint the wide range of genes responsible for fat deposition. Most studies have addressed the mechanism of fat deposition in the tail of fat-tailed sheep breeds [3,24,31,32,33,34], with the majority of them focusing on one gene, especially Leptin (*LEP*) [35,36,37], Fatty Acid Banding Protein4 (*FABP4*) [38,39,40], Adiponectin, C1Q And Collagen Domain Containing (*ADIPOQ*) [11,30], and Stearoyl-CoA Desaturase (*SCD*) [41,42]. Nowadays, instead of examining single genes, the whole genome of animals can be examined using next-generation sequencing (NGS) technologies. Using this approach, the whole transcriptomes of single cells can be examined using RNA sequencing (RNA-Seq). This method makes it possible to measure the expression of countless genes simultaneously and gives us a lot of information about the genome, even if there is little consensus on the obtained results, with the differentially expressed genes (DEGs) of one study not being supported by the results of another. The observed differences in the identified DEGs among studies requires a meta-analysis to uncover the genes that are responsible for fat deposition. Indeed, this method, through the use of rigorous statistical tests, can disclose patterns hidden in individual studies and allows one to draw conclusions with a high degree of reliability. For this study, by employing meta-analysis and machine learning approaches, we re-analyzed data from three recently conducted whole transcriptome RNA-seq studies of Guangling Large-Tailed and Small-Tailed Han sheep [11], Lori-Bakhtiari (fat-tailed) and Zel (thin-tailed) sheep [43], and Ghezel (fat-tailed) and Zel (thin-tailed) sheep [29]. The primary purpose of the current study was to identify differential meta-genes in male individuals of fat- and thin-tailed sheep breeds as transcriptomic signatures of fat deposition.

## 2. Materials and Methods

**Figure 1:**
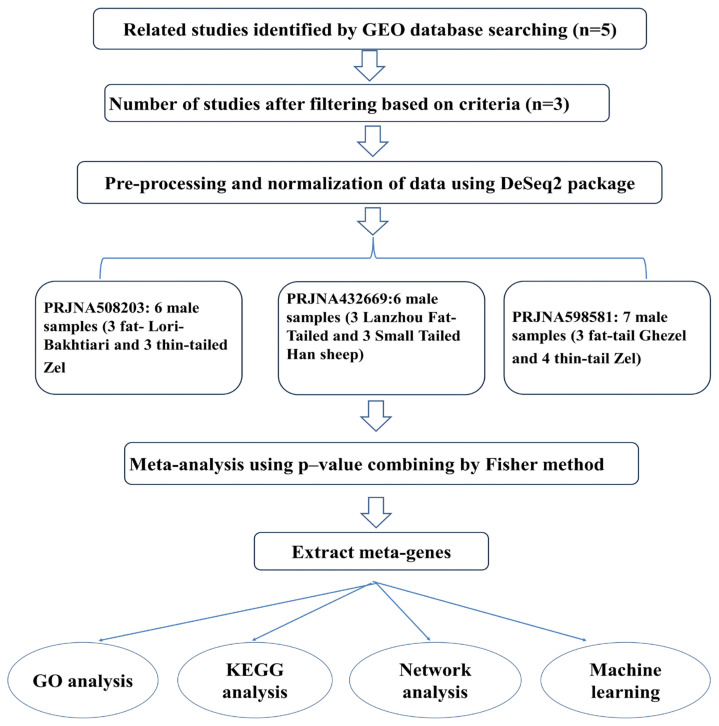
Flowchart of meta-analysis of the present study.

An overview of the process followed in this study is shown in Figure 1.

### 2.1. Dataset Collection

The keywords that were used were “Ovis aries”, “Fat-tailed”, “Thin-tailed”, “Fat deposition” and “Lipid metabolism”. We used PubMed Central (https://www.ncbi.nlm.nih.gov/pubmed accessed on 11 June 2021) and Google Scholar (https://scholar.google.com accessed on 11 June 2021). After identifying suitable RNA-seq studies of tail-fat deposition in the relevant fat- and thin-tailed sheep breeds, the related data were retrieved from either EMBL_EBI (https://www.ebi.ac.uk/arrayexpress accessed on 11 June 2021) or Gene Expression Omnibus (GEO) of NCBI (https://www.ncbi.nlm.nih.gov/gds accessed on 11 June 2021) databases. Two studies were excluded from our studies because the type of tissue examined or the type of RNA examined, or the sex of the samples were different. Finally, a set of sequencing data were collected from the fat tail tissue of male individuals from different sheep breeds.

### 2.2. Quality Control, Mapping, and Differential Gene Expression Analysis

Raw sequencing reads were subjected to quality control using FastQC (v0.11.5) [44] and trimmed using Trimmomatic software (v0.35); raw reads with adapter contamination and more than 10% of unknown bases, as well as with more than 50% of low-quality bases were trimmed out. Moreover, undesirable reads after trimming were filtered out [45]. The clean reads were mapped to the sheep reference genome v4.0 (ftp://ftp.ncbi.nlm.nih.gov/genomes/Ovis_aries/ accessed on 11 June 2021) using TopHat (v2.1.1) [46]. Sorted Binary Alignment Map (BAM) files were converted to *Sequence Alignment Map* (SAM) files, and count matrices were generated using htseq-count [47]. Then, the expression of the genes was normalized for library size and gene length to determine gene abundances using Fragment Per Kilo bases per Million (FPKM) [48], and the differentially expressed genes between the fat- and thin-tailed samples were identified using the DEseq2 package of R software [49]. For every dataset, each of these steps was carried out separately.

### 2.3. Meta-Analysis

The results of multiple scientific studies can be combined via a meta-analysis [50]. In addition to providing estimates of unknown effect sizes, meta-analyses can identify interesting and otherwise undetected relationships based on these results [51]. A set of *p*-values was computed for all three individual study datasets and later combined using the Fisher method of the meta RNAseq package [52]. Significance was set at *p* < 0.05. We named the final set of DEGs identified via our meta-analysis meta-genes.

### 2.4. GO Classification and KEGG Pathway Analysis

Gene ontology analysis is used to describe the function of genes in organisms. The Database for Annotation, Visualization, and Integrated Discovery (DAVID) (https://david.ncifcrf.gov/ accessed on 11 June 2021) was utilized to identify the category of meta-genes in the Gene Ontology (GO) based on Biological Processes (BP), Cellular Components (CC), and Molecular Functions (MF). Furthermore, the Kyoto Encyclopedia of Genes and Genomes (KEGG) pathway analysis tool (http://www.genome.jp/kegg/ accessed on 11 June 2021) was used to detect the metabolic pathways that are enriched by the meta-genes. For the enrichment analysis, terms with *p* < 0.05 generated using the modified Fisher Exact test were set as the cutoff thresholds.

### 2.5. Protein–Protein Network and Module Analysis

Protein functional interactions and their systematic properties help to provide context in molecular biology systems. The STRING database (http://string-db.org accessed on 11 June 2021) integrates protein–protein interactions that include direct (physical) and indirect (functional) interactions [53]. To predict protein–protein interactions, the identified meta-genes were imported into the STRING database (v11.0) [54]. The functional modules were detected via clustering using the K-means algorithm. Also, the Cytoscape plugin cytoHubba (v3.7.2) was utilized to identify hub genes using the Maximal Clique Centrality (MCC) method [55]. The PPI networks were constructed based on co-expression, neighborhood interactions, text mining, gene fusion, and databases as interaction sources. Functional modules were defined in the constructed networks by clustering the K-means algorithm into three modules [56].

### 2.6. Validation of Hub Genes Using Machine Learning Algorithms

Machine learning (ML) is a subset of artificial intelligence (AI) that uses algorithms to automatically learn insights and identify patterns from data to make better decisions. Decision Tree (DT) is one of the simplest and best models in machine learning, the main purpose of which is to predict the value of the target variable by using learning simple decision rules deduced from data features [57]. To assess the effectiveness of hub genes in distinguishing between fat-tailed and thin-tailed sheep, meta-genes and their corresponding expression values were identified and subjected to gene selection using seven different weighting algorithms. Normalized data were used for the attribute weighting algorithms (AWs). A range between 0 and 1 was considered for all weights, with values closer to 1 indicating important attributes for one meta-gene. These algorithms include Uncertainty, Relief, Gain Ratio, Information Gain, Gini Index, Chi-square, and Rule [58]. Only meta-genes with a weighting value greater than 0.7 were selected for DT construction using four criteria—Information Gain, Information Gain ratio, Gini index, and Accuracy—along with the leave-one-out cross-validation (LOOCV) method. During this process, the initial dataset was divided into training and testing sets. One sample at a time was removed from the initial dataset and added to the testing set, while all the others remained in the training set [59].

## 3. Results

### 3.1. Sequencing Data Collection

For this study, a total of approximately 200 Giga bases of RNA-seq from three datasets were utilized, comprising 19 samples in total. Each of the three datasets was sequenced using the Illumina HiSeq 2000 platform, and information pertaining to the three datasets is listed in Table 1.

### 3.2. Meta-Analysis of RNA-Seq Data

A total of 136 meta-genes were identified. Fisher’s method of differential analysis identified 20 meta-genes in PRJNA432669, 2 in PRJNA598581, and 75 in PRJA508203, along with 39 that had not been previously identified in the individual analyses.

### 3.3. Functional Enrichment Analysis of Meta-Genes

The top 10 BP terms are shown in Table 2.

**Figure 2:**
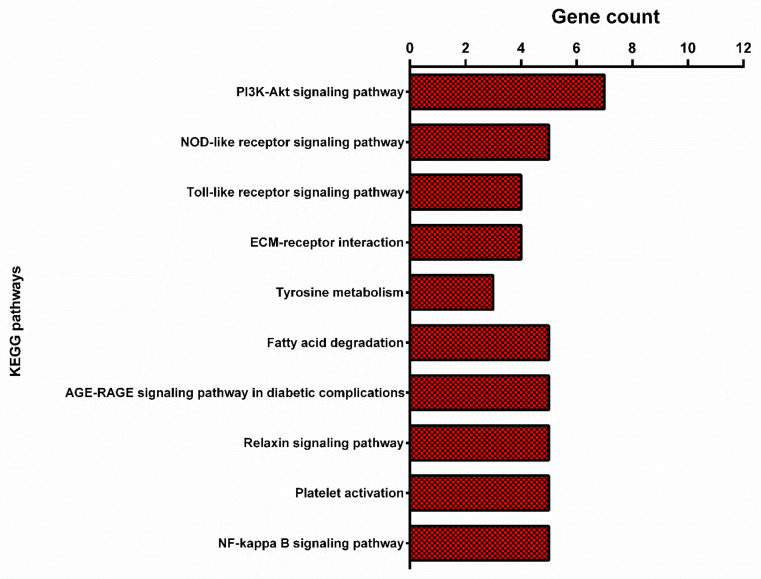
Top ten Kyoto Encyclopedia of Genes and Genomes (KEGG) pathways enriched by meta-genes.

The CC terms of “lipid droplet”, “Golgi lumen”, “endoplasmic reticulum lumen”, and MF terms of “lipoprotein lipase activity”, “dipeptidyl-peptidase activity”, “phospholipase activity”, “interleukin-17 receptor activity”, and “cAMP response element binding” were significantly enriched (*p* < 0.05). Several BP terms related to lipolysis, such as the “positive regulation of interleukin-1 beta secretion”, “positive regulation of interleukin-6 production”, “positive regulation of interleukin-8 production, “positive regulation of interleukin-10 production”, “regulation of interleukin-12 secretion”, “regulation of interleukin-13 secretion”, “positive regulation of interferon-gamma secretion”, and “positive regulation of tumor necrosis factor production” were enriched by meta-genes. The meta-genes were also mapped onto the KEGG pathway database to identify the pathways related to fat deposition (Figure 2).

### 3.4. Protein–Protein Interaction (PPI) Network and Module Analysis

**Figure 3:**
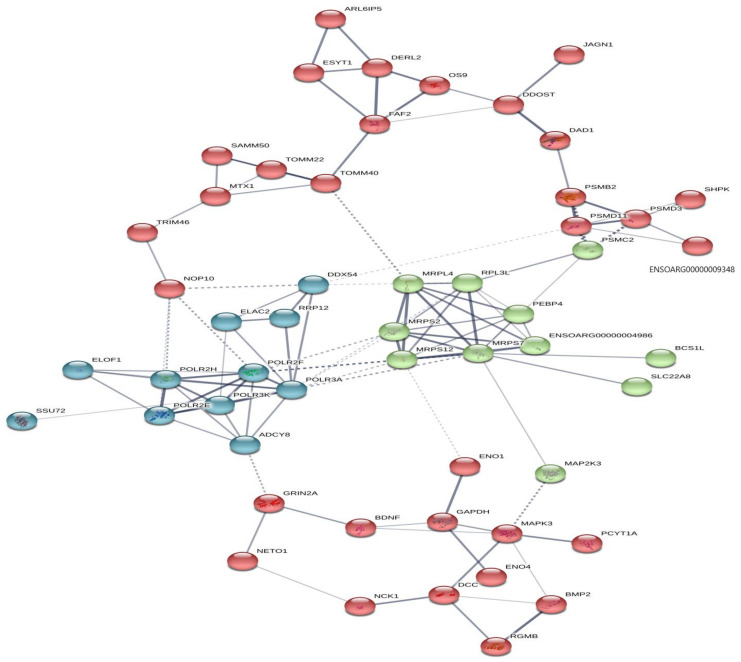
Protein–protein interaction (PPI) network and functional module analysis of meta-genes.

The PPI network of meta-genes revealed that 88% of the identified meta-genes had considerable interaction with the primary functional modules based on the confidence score of the interaction (confidence score < 0.7). In contrast, other disconnected nodes had no interaction in PPI networks (Figure 3). Also, TNF Receptor Associated Factor 6 (*TRAF6*) and Collagen, type I, Alpha 1 (*COL1A1*) meta-genes were identified as hub genes in PPI networks’ green and red modules, respectively.

### 3.5. Feature Selection for Machine Learning

**Figure 4:**
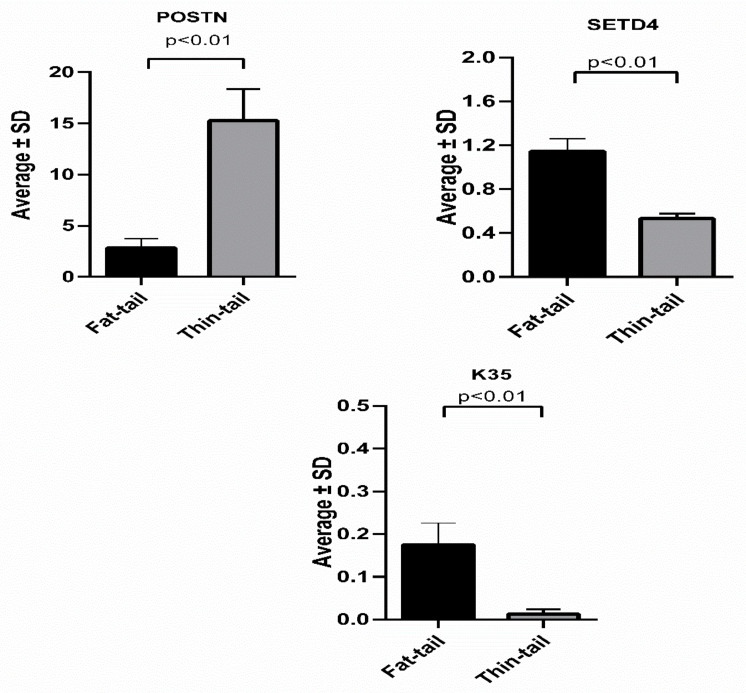
Three meta-genes with attribute weighting above 0.8. A two-sample *t*-test was used for the mean comparisons.

The meta-analysis resulted in the identification of 136 differentially expressed genes between the fat- and thin-tailed sheep breeds. Ten meta-genes, including Periostin (*POSTN)*, Keratin 35 (*K35)*, SET Domain Containing 4 (*SETD4*), Ubiquitin Specific Peptidase 29 (*USP29*), Ankyrin Repeat Domain 37 *(ANKRD37), ENSOARG00000001454*, Reticulon (*RTN2*), Proteoglycan (*PRG4*), and Leucine Rich Repeat Containing 4C (*LRRC4C*), were detected by the majority of the attribute weighting algorithms (with weight above 0.7) as the most informative genes. The top ten meta-genes in the discrimination of fat- and thin-tailed samples, confirmed by the majority of AWs (with an average weight above 0.7), are reported in Table 3. These meta-genes gained higher importance than the remaining meta-genes and were believed to be more effective in distinguishing the two breeds. According to Figure 4, a mean expression comparison between two types of breeds was carried out using a two-sample *t*-test. The expression of *POSTN*, *K35*, and *SETD4* meta-genes showed significant differences between the two sheep breeds.

The performances of the eight decision tree models are presented in Table 4. According to Table 4, among the decision tree models, the Random Forest with accuracy criterion and the Random Forest with gain_ratio criterion models surpassed the others in predicting fat deposition in both fat- and thin-tailed sheep breeds. These models had higher accuracy (above 0.85%).

**Figure 5:**
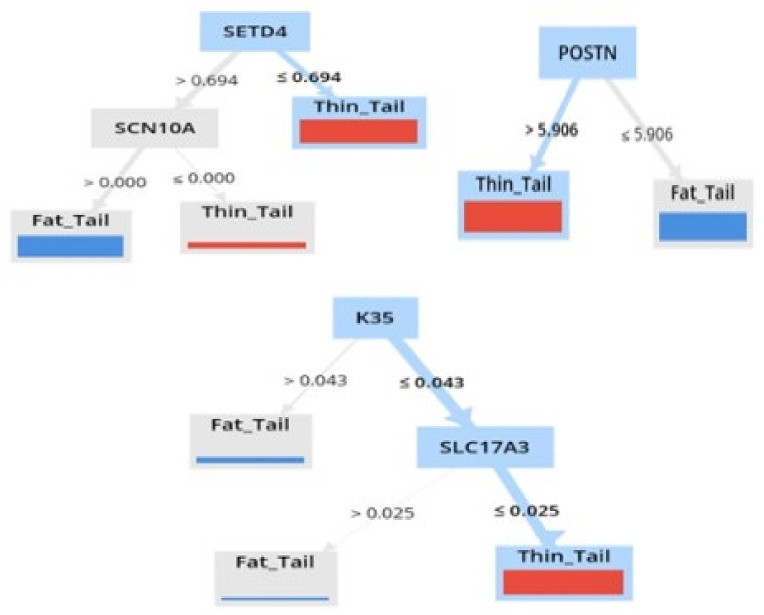
Decision tree induced by the Random Forest algorithm with gain_ratio criterion and accuracy criterion in distinguishing the fat-tailed sheep breeds from the thin-tailed sheep breeds in three meta-genes with attribute weighting above 0.8.

Also, the machine learning results show that the two meta-genes with the highest weight (*K35* and *SETD4*) were down-regulated, and *POSTN* was up-regulated in the thin-tailed sheep breeds compared to the fat-tailed sheep breeds (Figure 5).

## 4. Discussion

The current study identified genes that are informative in terms of the tail fat deposition of fat-tailed sheep breeds through using, for the first time in the literature, a machine learning approach. “ERK1 and ERK2 cascade” and “stress-activated MAPK cascade” terms are related to lipid metabolism. The two extracellular signal-regulated kinases (ERKs), ERK1 and ERK2, are members of the mitogen-activated protein kinase (MAPK) pathway and participate in both cell differentiation and proliferation, as well as the regulation of lipolysis [60]. ERK activation leads to the fast stimulation of hormone-sensitive lipase (HSL) activity and contributes to increased lipolysis. Documented pieces of evidence show that, as a stimulator of lipolysis, catecholamines cannot only activate cAMP-dependent protein kinase (PKA) but also activate ERKs of the MAPK pathway [61]. Five genes were enriched in the “ERK1 and ERK2 cascade”, including Kinase Insert Domain Receptor (*KDR*) or Vascular Endothelial Growth Factor Receptor 2 (*VEGFR2*), Galectin-9 (*LGALS9*), *TRAF6*, Nucleotide Binding Oligomerization Domain Containing 2 (*NOD2*), and Vascular Endothelial Growth Factor Receptor 3 (*VEGFR3*). Three of them (i.e., *KDR*, *NOD2*, and *LGALS9*) are closely related to lipid metabolism. A recent study showed that the *KDR* gene protects mice from obesity via fat burning and progressing lipolysis and enhancing basal metabolic rate [62]. In addition, it has been shown that galectin-9 enhances the production of microglial Tumor Necrosis Factor (TNF), which is the main lipolytic factor [63]. *NOD2* has also been shown to protect mice against diet-induced obesity and metabolic dysfunction, with obese mice lacking the *NOD2* gene suffering from metabolic dysfunction, including blood lipids, hyperglycemia, and steatosis, and the mass of adipose tissue and large fat droplets in liver cells increases [64]. These results were consistent with those of a recent study on the difference in adipose tissue metabolic pathways in fat- and thin-tailed sheep breeds [34].

There is a direct connection between lipid metabolism and terms such as Interleukin-1 (*IL-1*) [65], Interleukin-6 (*IL-6*) [29], Interleukin-8 (*IL-8*) [66], Interleukin-10 (*IL-10*) [67], Interleukin-12 (*IL-12*) [68]. The “positive regulation of tumor necrosis” factor production is also one of the BP terms closely related to lipolysis. It has been reported that, in human adipocytes, TNF-α stimulates lipolysis via the elevation of intracellular cAMP, MAPK, and extracellular signal-related kinase (ERK) [69,70]. All the inflammatory pathways and the lipolytic ERK/MAPK/TNF pathways enhance lipolysis. In other words, these pathways improve pro-inflammatory cytokine expression and increase lipolytic activity. Finally, these results suggest that some essential lipolytic pathways (e.g., “MAPK signaling pathway” and “TNF signaling pathway”) and inflammatory pathways (e.g., “positive regulation of *IL-1* secretion”, “positive regulation of *IL-6* production”, “positive regulation of *IL-8* production”, and “positive regulation of *IL-10* production”) are active in thin-tailed sheep breeds. There have been reports that *IL-1* and interferon-gamma (IFN) stimulate lipolysis in cultured adipocytes [71]. In addition, inflammatory cytokine *IL-6* and *IL-8* mRNA expressions are involved in lipopolysaccharide-induced lipolysis in human adipocytes [67]. Another study introduced *IL-6* as a hub gene in the fat lipolysis of thin-tailed sheep breeds [29]. This gene is well known to be a lipolytic factor that stimulates fat lipolysis and fatty acid oxidation in humans [72,73], dairy cows [74], rats [75], mice [76,77], and sheep [29]. Thus, the up-regulation of the aforementioned genes in thin-tailed sheep might be closely related to lipolysis.

Our KEGG pathway analysis of the meta-genes revealed significant pathways (adjusted *p*-value < 0.05). Recent findings show that a set of pathways, such as lipid metabolism, extracellular matrix (ECM) remodeling, molecular transport, and inflammatory response, are enriched by a set of functional genes that maintain lipid homeostasis in response to extreme environments in tailed animals [34]. In the present study, some pathways, including “fatty acid degradation”, “NF-kappa B signaling pathway”, “NOD-like receptors”, and “Toll-like receptors”, were all related to fat metabolism as an inflammatory response. One of the significant terms in thin-tailed sheep breeds compared with fat-tailed sheep breeds is the “fatty acid degradation” pathway, the pathway known for the lipolysis of adipocytes. Another considerable term is “NF-kappa B signaling pathway”. NF-κB is important for TNF-α-induced lipolysis of adipose tissue. Tumor necrosis factor-α (TNF-α) increases lipolysis in adipose tissue via the MAPK pathway. Several meta-genes, including Myeloid Differentiation Primary Response 88 (*MYD88*), TGF-Beta Activated Kinase 1 (MAP3K7) Binding Protein 2 (*TAB2*), Interleukin-1 Receptor-associated Kinase 1 (*IRAK1*), Phospholipase C Gamma 2 (*PLCG2*), and *TRAF6*, were found to be enriched in the “NF-kappa B signaling pathway”, which is closely related to lipid metabolism.

“ECM-receptor interaction” is another significant pathway that is central to adipogenesis and fat tissue architecture [78]. Fat accumulation is an inflammatory condition related to increased extracellular matrix gene expression [34,79]. However, a direct connection between ECM gene expression and fat tissue inflammation has not been reported. In a recent study, transcriptome analysis of two broiler chickens showed that the extracellular matrix receptor interaction signaling pathway is crucial to chicken meat quality. This pathway might change intramuscular fat content, affecting broiler meat flavor [80]. In another study, comparative transcriptome analysis of three adipose tissues (i.e., subcutaneous, intramuscular, and omental adipose tissue) showed that the interactions between transmembrane receptors of fat cells and ECM components depend on depot-specific adipogenesis [81]. Cell adhesion receptors and ECM components interact with each other, creating a complex network. According to one study, cell surface receptors receive signals from the ECM that influence growth, survival, migration, differentiation, and proliferation in maintaining cell homeostasis [82]. All enriched meta-genes in this pathway are up-regulated in thin-tailed sheep breeds, possibly due to the interaction between ECM components. This result is in accordance with a recent study that showed that fat-tailed sheep are less responsive to seasonal changes in inflammation and fat cell size, ECM regeneration, and lipid metabolism, which indicates the improvement of homeostasis [34].

“NOD-like receptors” (NLR) and “Toll-like receptors” (TLR) are two pattern recognition receptors that have severe roles in the inflammation of adipocytes and the immune response [83]. Both of the mentioned KEGG pathways were significantly enriched in the current study. The activation of a sub-family of these receptors [84,85,86] has been shown to stimulate lipolysis from adipose tissue or adipocytes. Therefore, all these pathways maintain fat homeostasis in response to extreme environments in sheep breeds.

The meta-analysis results revealed 39 meta-genes that were insignificant in each of the individual studies, indicating the higher statistical power of the meta-analysis in the discovery of biosignatures. In addition, the results derived from using machine learning showed that three significant genes (*POSTN*, *K35*, and *SETD4*) gained higher weights (>0.8) than others, according to the AW algorithms. Interestingly, these meta-genes, along with other genes with a weight >0.8, are associated with lipid metabolism. The decision tree induced by the Random Forest Model shows that *POSTN*, *K35*, and *SETD4* meta-genes directly affect lipid metabolism. Interestingly, the *SETD4* gene is one of the 39 meta-genes that were insignificant in the individual studies. Currently, the role of the *K35* gene in fat metabolism has not been identified, but two other genes have been shown to be related to fat metabolism. There have been reports that the *SETD4* gene has considerable potential for tumorigenesis. It is thought that the *SETD4* gene has proliferation potential in fat cells [87]. Thus, lipogenesis might be associated with the up-regulation of the *SETD4* gene in fat-tailed sheep. Moreover, the loss of *POSTN* attenuates lipid metabolism in adipose tissue [88]. Among the decision tree models, both the Random Forest with accuracy criterion and the Random Forest with gain_ratio criterion models outperformed others in the prediction of fat deposition in sheep breeds. These high-performance models enabled us to detect the *POSTN*, *K35*, and *SETD4* meta-genes as biosignatures or biomarkers for fat metabolism. Therefore, the combination of meta-analysis and machine learning approaches employed in the current study improved the power of discovering informative genes that may aid the progress of animal breeding strategies to optimize tail fat in fat-tailed breeds.

## 5. Conclusions

Fat deposition is a complex trait that requires comprehensive research to be elucidated. However, the integration of machine learning and meta-analyses approaches, as carried out for the current work, may help to better understand the most critical causal genes that can be exploited as strong biomarkers of fat deposition; in our study, three meta-genes, namely, *POSTN*, *K35*, and *SETD4*, were identified as strong biosignatures of fat deposition. Our findings may provide a base for strategies to optimize fat deposition in the tail of fat-tailed breeds, thus decreasing the fat content of carcasses.

## Figures and Tables

**Table 1:** Summary information of three RNA-seq datasets sourced from sheep tail fat tissue.

GEO Accession Number	Number of Samples		Tissue Sample	Age of Slaughter (Month)	Read Length	Reference
	Thin-Tailed	Fat-Tailed				
PRJNA432669	3	3	Tail	6	150 bp	[11]
PRJNA508203	3	3	Tail	6	150 bp	[43]
PRJNA598581	4	3	Tail	6	150 bp	[29]

**Table 2:** Top 10 Biological Process terms enriched by meta-genes.

Biological Process Terms	Adjusted *p*-Value
Positive regulation of T cell cytokine production	0.002
Extracellular matrix organization	0.004
Stress-activated MAPK cascade	0.008
ERK1 and ERK2 cascade	0.008
Positive regulation of interleukin-10 production	0.009
Positive regulation of interleukin-1 secretion	0.016
Positive regulation of interleukin-6 production	0.034
Positive regulation of interleukin-8 production	0.036
Positive regulation of interferon-gamma (IFN)secretion	0.04
Positive regulation of tumor necrosis factor (TNF) production	0.04

**Table 3:** Top 10 out of the 136 meta-genes according to seven attribute weighting algorithms (AWs).

Attribute	Weight_Info Gain Ratio	Weight_Rule	Weight_Chi Squared	Weight_Gini Index	Weight_Uncertainty	Weight_Relief	Weight_Info Gain	Average_Weight
*POSTN*	1	1	0.5	0.8	0.6	0.6	0.8	0.8
*K35*	0.8	0.9	0.6	0.6	0.7	1	0.6	0.8
*SETD4*	0.8	1.0	0.7	0.7	0.6	0.9	0.6	0.8
*USP29*	0.7	0.8	0.8	0.5	0.7	1.0	0.5	0.7
*ANKRD37*	0.7	0.9	0.8	0.5	0.7	0.9	0.5	0.7
*ENSOARG00000001454*	0.8	1	0.5	0.6	0.4	0.7	0.6	0.7
*RTN2*	0.7	1	0.6	0.5	0.6	0.8	0.5	0.7
*PRG4*	0.8	1	0.3	0.6	0.4	0.9	0.6	0.7
*LRRC4C*	0.7	0.8	0.6	0.5	0.5	1	0.5	0.7

**Table 4:** Performances of machine learning models in the distinction of fat- and thin-tailed sheep breeds via ten-fold cross validation.

Model	Accuracy
Random Forest with accuracy criterion	90% +/− 22.36%
Random Forest with gain_ratio criterion	85% +/− 13.69%
Decision Tree with gain_ratio criterion	58.33% +/− 37.27%
Decision Tree with accuracy criterion	75% +/− 35.36%
Deep Learning with Tanh parameter	85% +/− 22.36%
Deep Learning with Rectifier parameter	75% +/− 25.00%
Deep Learning with Maxout parameter	56.67% +/− 18.07%
Naïve Bayes	78.33% +/− 21.73%

## Data Availability

All used data are publically available. The accession numbers have been reported in Table 1.
